# Complete mitochondrial genome of the spiny rock crab *Thalamita crenata* (rüppell, 1830) (Crustacea: Decapoda: Portunidae) from China coast and its phylogeny

**DOI:** 10.1080/23802359.2018.1508384

**Published:** 2018-10-30

**Authors:** Zhuofang Xie, Tinghe Lai, Khor Waiho, Huaqiang Tan, Hongyu Ma

**Affiliations:** a Guangdong Provincial Key Laboratory of Marine Biotechnology, Shantou University, Shantou, China;; b STU-UMT Joint Shellfish Research Laboratory, Shantou University, Shantou, China;; c Qinzhou University, Ocean College, Qinzhou, China;; d Guangxi Academy of Oceanography, Nanning, China

**Keywords:** Thalamita crenata, mitochondrial genome, phylogeny, evolutionary relationships

## Abstract

To understand the evolution of the swimming crab *Thalamita crenata*, the complete mitochondrial genome of *T. crenata* from China was sequenced and analyzed. The circular mitogenome sequence was 15,787 bp in length, made up of 13 protein-coding genes, 22 transfer RNA genes, two ribosomal RNA genes and a control region. The overall mitogenome composition was 34.40% for A, 11.55% for G, 35.31% for T, and 18.74% for C, respectively, with a high A + T content of 69.71%. Phylogenetic analysis showed that *T. crenata* was closest to the genus *Charybdis*.

The spiny rock crab, *Thalamita crenata*, under the Family Portunidae is mainly distributed in Western Pacific and Indian Ocean (Dai et al. 1986; Dai and Yang 1991; Stephenson 1972). It inhabits the rocky and muddy intertidal regions of mangrove forests (Vezzosi et al. 1994). Unlike other big-size economic portunids, such as the mud crab *Scylla paramamosain* and the red crab *Charybdis feriatus*, *T. crenata* has less economic value (Manickaraja and Balasubramanian 2009). However, its natural population is under threat from local fishing pressure (Manickaraja and Balasubramanian 2009). In order to gain a deep understanding of the phylogeny and species evolution, we described, in this study, the complete mitochondrial genome of *T. crenata* from China.

Specimens of *T. crenata* were collected from Weizhou Island (21.0234°N, 109.0940°E), Guangxi province, China and preserved in pure ethanol in Marine Biology Institute of Shantou University. Total genomic DNA was extracted from muscle tissues and the complete mitogenome sequence was obtained by long and conventional PCR. Additionally, the complete mitochondrial genomes of 17 brachyuran species and one shrimp (*Hapiosquilla harpax*) were downloaded from NCBI. The ND6 gene showed a high degree of heterogeneity and caused poor phylogenetic performance (Miya and Nishida 2000), so it was omitted in next analysis. The maximum-likelihood (ML) phylogenetic tree was constructed by MEGA7 software based on 12 protein-coding genes.

The complete mitochondrial genome of *T. crenata* was 15,787 bp in length (GenBank accession number: MH425338), including 13 protein-coding genes, 22 transfer RNA genes, two ribosomal RNA genes, and a control region. The overall mitogenome composition was 34.40% for A, 11.55% for G, 35.31% for T, and 18.74% for C, respectively, with a high A + T content of 69.71%. The phylogenetic analysis showed that *T. crenata* was closest to the genus *Charybdis* (Figure 1). The control region (D-loop) was 898 bp long and between the genes tRNA^Pro^ and tRNA^Phe^, with a a high similarity of 95% to the corresponding region of *T. crenata* collected from Australia (Tan et al. 2016).

**Figure 1.**
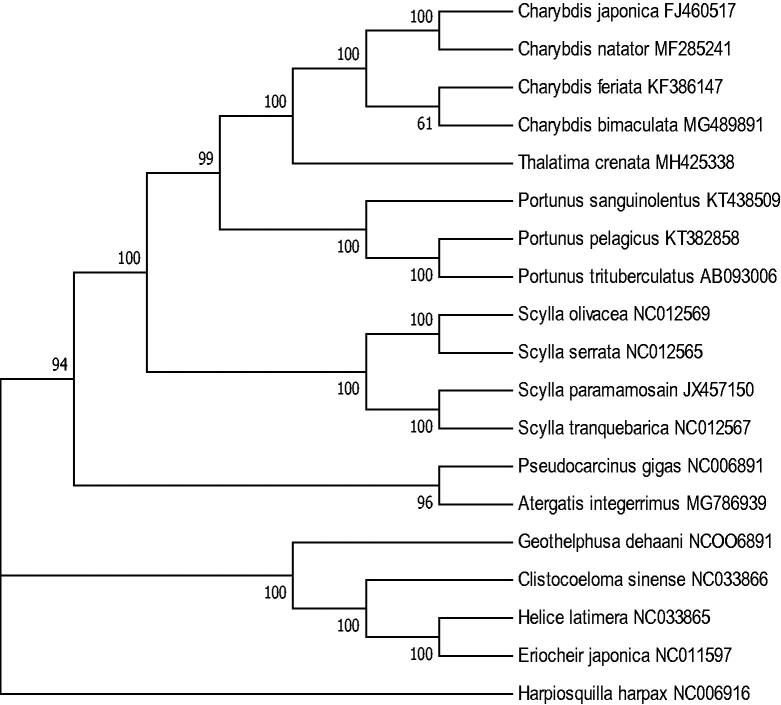
Molecular phylogeny of *T. crenata* and other related species based on 12 protein-coding genes. The complete mitochondrial genomes of 18 species were downloaded from NCBI, with *Hapiosquilla harpax* as an outgroup.

## References

[CIT0001] Dai AY, Yang SL, Song YZ 1986 Marine crabs in China Sea. Beijing Marine Publishing Company; p.194–196.

[CIT0002] Dai A, Yang SL 1991 Crabs of the China seas. Beijing: China Ocean Press; p.1–74.

[CIT0003] Manickaraja M, Balasubramanian TS 2009 Occurrence of the deep sea crab, *Thalamita crenata* in shallow water gillnet (mural valai) operation at Tharuvaikulam, north of Tuticorin. Mar Fish Inf Serv Tech Ext Ser. 201:28.

[CIT0004] Miya M, Nishida M 2000 Use of mitogenomic information in teleostean molecular phylogenetics: a tree-based exploration under the maximum-parsimony optimality criterion. Mol Phylogenet Evol. 17:437–455. 11133198 10.1006/mpev.2000.0839

[CIT0005] Stephenson W 1972 Portunid crabs from the ludo-West Pacific and Western America in the zoologica. Museum Copenhagen (Decapoda, Brachyura, Portunidae). Steenstrupia. 2:127–156.

[CIT0006] Tan MH, Gan HM, Lee YP, Austin CM 2016 The complete mitogenome of the swimming crab *Thalamita crenata* (Rüppell, 1830) (Crustacea; Decapoda; Portunidae). Mitochondr DNA. 27:1275–1272. 10.3109/19401736.2014.945553 25090400

[CIT0007] Vezzosi R, Barbaresi S, Anyona D, Vannini M 1994 Activity patterns in *Thalamita crenata* Portunidae, decapoda: a shaping by the tidal cycles. Mar Behav Physiol. 24:207–214.

